# Efficacy of Antiresorptive Drugs on Bone Mineral Density in Post-Menopausal Women With Early Breast Cancer Receiving Adjuvant Aromatase Inhibitors: A Systematic Review of Randomized Controlled Trials

**DOI:** 10.3389/fonc.2021.829875

**Published:** 2022-01-21

**Authors:** Alessandro de Sire, Lorenzo Lippi, Konstantinos Venetis, Stefania Morganti, Elham Sajjadi, Claudio Curci, Antonio Ammendolia, Carmen Criscitiello, Nicola Fusco, Marco Invernizzi

**Affiliations:** ^1^ Department of Medical and Surgical Sciences, University of Catanzaro “Magna Graecia”, Catanzaro, Italy; ^2^ Physical and Rehabilitative Medicine, Department of Health Sciences, University of Eastern Piedmont “A. Avogadro”, Novara, Italy; ^3^ Division of Pathology, IEO, European Institute of Oncology IRCCS, Milan, Italy; ^4^ Department of Oncology and Hemato-Oncology, University of Milan, Milan, Italy; ^5^ Division of Early Drug Development, IEO, European Institute of Oncology IRCCS, Milan, Italy; ^6^ Physical Medicine and Rehabilitation Unit, Department of Neurosciences, ASST Carlo Poma, Mantova, Italy; ^7^ Translational Medicine, Dipartimento Attività Integrate Ricerca e Innovazione (DAIRI), Azienda Ospedaliera SS. Antonio e Biagio e Cesare Arrigo, Alessandria, Italy

**Keywords:** breast cancer, early breast cancer, bone health, quality of life, osteoporosis, rehabilitation

## Abstract

**Background:**

Cancer treatment-induced bone loss (CTIBL) is a frequent complication of breast cancer therapies affecting both disability and health-related quality of life (HRQoL). To date, there is still a lack of consensus about the most effective approach that would improve bone health and HRQoL. Therefore, the aim of this systematic review of randomized controlled trials (RCTs) was to summarize the evidence on the effects of antiresorptive drugs on CTIBL in patients with early breast cancer.

**Methods:**

PubMed, Scopus, and Web of Science databases were systematically searched up to April 30, 2021 to identify RCTs satisfying the following PICO model: P) Participants: postmenopausal women with early breast cancer receiving adjuvant aromatase inhibitors (AI), age >18 years; I) Intervention: antiresorptive drugs (i.e. bisphosphonates and/or denosumab); C) Comparator: any comparator; O) Outcome: bone mineral density (BMD) modifications. Moreover, a quality assessment was performed according to the Jadad scale.

**Results:**

Out of the initial 2415 records, 21 papers (15 studies) were included in the data synthesis. According to the Jadad scale, 6 studies obtained a score of 5, 1 study obtained a score of 4, 13 studies obtained a score of 3, and 1 study with score 1. Although both bisphosphonates and denosumab showed to increase BMD, only denosumab showed significant advantages on fractures.

**Conclusions:**

Bone health management in patients with early breast cancer receiving adjuvant AIs remains challenging, and the optimal therapeutic approach is not standardized. Further studies are needed to investigate CTIBL, focusing on both the need for antiresorptive drugs and their duration based on individual patients’ characteristics.

**Systematic Review Registration:**

https://www.crd.york.ac.uk/prospero, identifier CRD42021267107.

## Introduction

Breast cancer (BC) is the most prevalent malignancy in women worldwide, with incidence increasing in last decades (1). Oppositely, mortality from BC decreased in last years, due to the significant advancements in screening programs and therapeutical interventions (2). In response to the progressive increase of women living after a diagnosis of BC, survivorship issues related to cancer treatment and its impact on bone health and health-related quality of life (HRQoL) have progressively emerged (3–9).

Cancer treatment-induced bone loss (CTIBL) is a frequent side effect of the pharmacotherapy used for treating BC. While chemotherapy might lead to an unspecific increase in bone resorption, hormone therapies (HT) reduce residual serum endogenous estrogen levels, with a consequent decrease in bone mineral density (BMD) and an increase in fragility fracture risk (10–17). To date, aromatase inhibitors (AI) are considered the gold standard adjuvant therapy for postmenopausal women with hormone receptor (HR)-positive early BC (EBC) (18, 19). In such patients, a significant decrease in bone density has been observed (20, 21). To counter bone loss induced by AIs in BC patients, several anti-resorptive molecules have been investigated (22, 23). The ZO-FAST study supported the efficacy of zoledronic acid in increasing BMD in postmenopausal women receiving adjuvant AIs (24). In addition, the ABCSG-12 trial showed that zoledronic acid along with endocrine therapy could also increase disease-free survival (DFS) in premenopausal women with EBC (25). In 2015, the Early Breast Cancer Trialists’ Collaborative Group (EBCTCG) published a meta-analysis of individual patient data investigating bisphosphonates (BPs) in the adjuvant setting of EBC, including data from 18,766 women in 26 trials. All tumor subtypes and adjuvant treatments were considered. Use of BPs reduced both bone recurrence (rate ratio [RR] 0.83; p=0.004) and bone fractures (RR: 0.85; p=0.02), with a significant impact also on distant recurrence (RR 0.92; p=0.03) and BC mortality (RR 0.91; p=0.04). Notably, the subgroup analysis showed how the added value of bisphosphonate is limited in premenopausal patients, while postmenopausal patients derived a greater benefit in all outcomes.

Denosumab, a fully human IgG2 monoclonal antibody, has been proposed to treat CTIBL in BC patients undergoing HT not only by improving BMD but also by reducing the rate of clinical fragility fractures (both hip and vertebrae) (12, 26, 27).

Although the long-term management of bone health in BC patients through the combination of different pharmacological therapies is gaining interest, most studies conducted to date have only assessed the effects of a single drug in terms of BMD improvement or fracture risk reduction (28–30). Thus, the gap of knowledge about tailored and effective bone health interventions is far from being understood.

Therefore, this systematic review aims to summarize the current evidence on the efficacy of anti-resorptive agents and their impact on bone health and HRQoL in post-menopausal patients with EBC receiving adjuvant AIs.

## Materials and Methods

### Study Registration

This systematic review of randomized controlled trials (RCTs) has been performed ethically in accordance with the Preferred Reporting Items for Systematic Reviews and Meta-analyses (PRISMA) statement (31). The PRISMA Checklist is provided as Supplementary Material. A protocol was developed before study initiation and submitted to PROSPERO (https://www.crd.york.ac.uk/prospero; registration number CRD42021267107).

### Search Strategy

We systematically searched PubMed/Medline, Scopus, and Web of Science for RCTs published up to April 30, 2021. Two investigators independently searched the databases. The search strategy is reported in **Table 1**.

**Table 1:** Search strategy.

***PubMed*** ((Breast cancer[Title/Abstract]) OR Breast cancer [MeSH Terms]) OR ((aromatase inhibitors [Title/Abstract]) OR aromatase inhibitors [MeSH Terms]) AND (((((osteoporosis [Title/Abstract]) OR bisphosphonate[Title/Abstract]) OR zoledronic acid[Title/Abstract]) OR Denosumab[Title/Abstract]) OR ((((osteoporosis [Title/Abstract]) OR bisphosphonate[MeSH Terms]) OR zoledronic acid[MeSH Terms]) OR Denosumab [MeSH Terms])) AND (((((fracture [Title/Abstract]) OR bone mineral density [Title/Abstract]) OR pain [Title/Abstract]) OR HRQoL [Title/Abstract]) OR ((((fracture [MeSH Terms]) OR bone mineral density [MeSH Terms]) OR pain [MeSH Terms]) OR HRQoL [MeSH Terms]))
***Scopus*** TITLE-ABS-KEY (breast cancer AND aromatase inhibitors AND (osteoporosis OR bisphosphonate OR zoledronic acid OR Denosumab) AND (fracture OR bone mineral density OR pain OR HRQoL)
***Web of Science*** (breast cancer AND aromatase inhibitors AND (osteoporosis OR bisphosphonate OR zoledronic acid OR Denosumab) AND (fracture OR bone mineral density OR pain OR HRQoL))

### Selection Criteria

In accordance with the PICO model (32), we considered eligible RCTs satisfying the following criteria:

P) Participants: postmenopausal women with early BC receiving adjuvant AI, age >18 years;
I) Intervention: antiresorptive drugs (i.e. BPs and/or denosumab);
C) Comparator: any comparator;
O) Outcome: BMD modifications.

Only RCTs published in International journals in English language were included. The exclusion criteria were: i) studies involving animals; ii) language other than English; iii) participants with pregnancy; iv) cancer different of BC; v) studies involving patients with metastatic BC; vi) conference abstracts.

After duplication removal, two investigators independently reviewed the title and abstracts of retrieved articles to choose relevant articles. A third reviewer was asked in case of disagreement.

### Data Extraction and Synthesis

Data were assessed and extracted from full-text documents by two independent reviewers (AdS and LL). Any disagreement was solved by discussion or consulting a third reviewer (MI).

The following data were extracted: 1) title and trial name; 2) authors; 3) publication year; 4) number of patients included; 5) intervention characteristics; 6) comparator arm(s); 7) bone-health related outcomes; 8) follow-up.

A descriptive approach was used to synthesize both study characteristics and data extracted. Subgroup analysis has been performed based on the specific drug assessed in the studies included.

### Study Quality and Risk of Bias

Study quality was assessed according to the Jadad scale by two reviewers independently (33). In case of disagreement, a third reviewer was involved in the decisional process to achieve consensus. The clinical trials with a Jadad score between 3 and 5 points were considered as high-quality studies.

## Results

### Main Characteristics of the Included Studies

**Figure 1:**
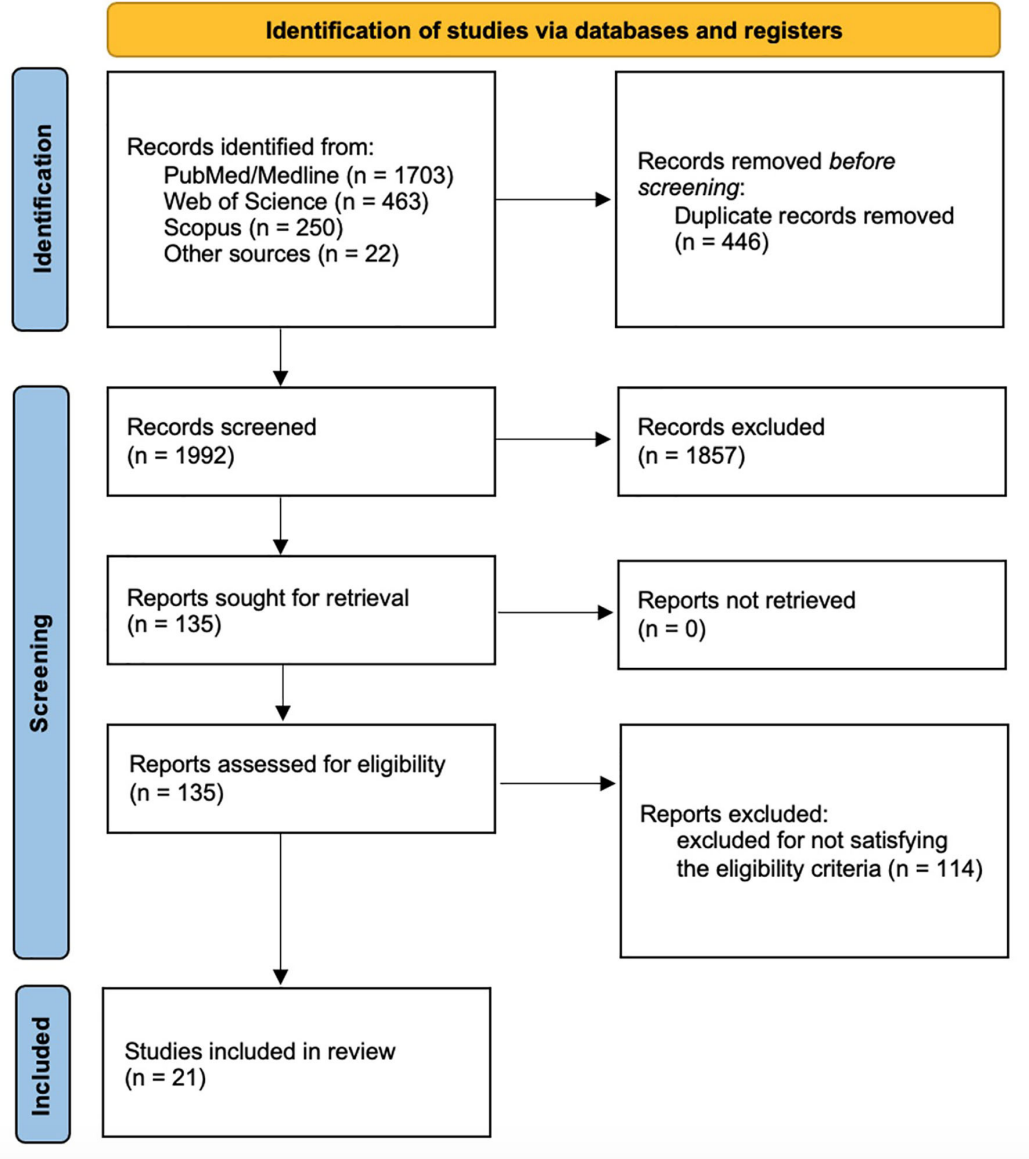
PRISMA flow diagram.

A total of 2416 records were identified from the search process (PubMed/Medline: 1703 records; Web of Science: 463 records; Scopus: 250 records) and 22 records were identified by reference lists of primary studies. After duplication removal, 1992 records were screened for title and abstract. Therefore, 1857 records were excluded, and 135 full-text studies were screened. One hundred and seventeen records were excluded for not satisfying the eligibility criteria. Finally, the following 21 papers (15 RCTs) were included in the present systematic review: Livi (2019) (29), Gnant (2015) (34), Gnant (2019) (35), Hines (2009) (36), Wagner-Johnston (2015) (37), Greenspan (2015) (38), Coleman (2013) (39), Rhee (2013) (40), Lester (2008) (41), Lester (2012) (42), Takahashi (2012) (43), Llombart (2012) (44), Van Poznak (2010) (45), Markopoulos (2010) (46), Eidtmann (2010) (47), Brufsky (2009) (48), Ellis (2008) (49), Bundred (2008) (24), Brufsky (2008) (50), Brufsky (2012) (51), Safra (2011) (52). Further details on the identification and inclusion/exclusion of the screened studies are reported in **Figure 1**.

Main characteristics of the 15 clinical trials (21 papers) included (24, 29, 34, 35, 39–44, 46–52) are summarized in **Table 2**. These RCTs were published between 2008 (24, 41, 49, 50) and 2019 (29, 35). Most of them (7; 46.7%) were International collaborations (24, 34, 35, 39, 44, 45, 47–51), whereas 3 studies were carried out in Europe (1 in the United Kingdom (41, 42), 1 in Italy (35), 1 in Greece (46)), 3 in Asia (1 in Japan (43), 1 in Korea (40), 1 in Israel (52)) and 2 in the USA (36–38).

**Table 2:** Main characteristics of the articles included in the present systematic review.

Authors	Journal	Publication year	Nationality	Population	Age (years)	Hormonal therapy	Intervention	Comparator	Outcomes	Follow-up
	***Alendronate***									
Rhee et al. (40)	*Endocr J*	2013	Korea	n: 98 IG: 49 CG: 49	IG: 57.1 ± 1.0 CG: 58.5 ± 1.1	Anastrozole or letrozole	Alendronate 5 mg + calcitriol 0.5 µg daily	Placebo	**- LS BMD** **- TH BMD** - Bone turnover biomarkers - safety	24 weeks
	***Denosumab***									
Ellis et al. (49) (NCT00089661)	*J Clin Oncol.*	2008	International Collaboration	n: 252 IG: 127 CG: 125	IG: 59.2 ± 8.9 CG: 59.7 ± 9.7	Anastrozole, letrozole, or exemestane	Denosumab 60 mg sc every 6 months	Placebo	**- LS BMD** - TH BMD - FN BMD - Radius BMD - Bone turnover biomarkers - Vertebral and nonvertebral fractures - Safety - Overall survival	24 months
Gnant et al. (34) (ABCSG-18)	*The Lancet*	2015	International Collaboration	n: 3420 IG: 1711 CG: 1709	64 (38 – 91)	Anastrozole, letrozole, or exemestane	Denosumab 60 mg sc every 6 months	Placebo	**- Time to first fracture** - Vertebral and nonvertebral fractures - LS BMD - TH BMD - FN BMD - Disease-free survival - Bone-metastasis free survival - Overall survival	36 months
Gnant et al. (35) (ABCSG-18)	*Lancet Oncol.*	2019	International Collaboration	n: 3420 IG: 1711 CG: 1709	64 (38 – 91)	Anastrozole, letrozole, or exemestane	Denosumab 60 mg sc every 6 months	Placebo	**- Time to first fracture** - Vertebral and nonvertebral fractures - LS BMD - TH BMD - FN BMD - Disease-free survival - Bone-metastasis free survival - Overall survival	96 months
	***Ibandronate***									
Lester et al. (41) (ARIBON)	*Clinical Cancer Research*	2008	UK	n: 50 IG: 25 CG: 25	IG: 67.8 (58.9-73.4) CG: 67.5 (63.6-71.0)	Anastrozole	Ibandronate 150 mg every month	Placebo	**- LS BMD** **- TH BMD** - Bone turnover biomarkers - Safety	24 months
Lester et al. (42) (ARIBON)	*Journal of Bone Oncology*	2012	UK	n: 50 IG: 25 CG: 25	IG: 67.8 (58.9-73.4) CG: 67.5 (63.6-71.0)	Anastrozole	Ibandronate 150 mg every month for 24 months	Ibandronate 150 mg every month started after 24 months	**- LS BMD** **- TH BMD**	60 months
Livi et al. (29) (BONADIUV)	*European Journal of Cancer*	2019	Italy	n: 144 IG: 89 CG: 82	IG: 60.5 (54.3-67.0) CG: 59.6 (53.9-68.0)	Anastrozole, letrozole, or exemestane	Ibandronate 150 mg every month	Placebo	**- LS BMD** **- TH BMD** - Safety - Disease recurrence - Overall survival	24 months
	***Risedronate***									
Greenspan et al. (38) (NCT00485953)	*Osteoporosis International*	2015	USA	n: 109 IG: 55 CG: 54	IG: 65 ± 1 CG: 64 ± 1	Anastrozole, letrozole, or exemestane	Risedronate 35 mg every week	Placebo	- **LS BMD** **- TH BMD** - FN BMD - TB BMD - Bone turnover biomarkers	24 months
Markopoulos et al. (46) (ARBI)	*Breast Cancer Research*	2010	Greece	n: 70 IG: 37 CG: 33	IG: 62.6 ± 8.5 CG: 64.5 ± 9.2	Anastrozole	Risedronate 35 mg every week	No treatment	- **LS BMD** **- TH BMD**	24 months
Van Poznak et al. (45) (SABRE)	*Journal of Clinical Oncology*	2010	International Collaboration	n: 154 IG: 77 CG: 77	IG: 63.8 CG: 64.8	Anastrozole	Risedronate 35 mg every week	Placebo	**- LS BMD** - TH BMD - Bone turnover biomarkers	24 months
	***Zoledronate***									
Brufsky et al. (52)	*The Oncologist*	2008	International Collaboration	n: 1667 IG: 833 CG: 834	IG: 58 (35-87) CG: 59 (37-89)	Letrozole	Immediate zoledronate 4 mg iv every 6 months	Delayed zoledronate 4 mg iv every 6 months	**- LS BMD** - TH BMD - Bone turnover biomarkers - Disease recurrence - Safety	12 months
Brufsky et al. (48) (Z-FAST)	*Clinical Breast Cancer*	2009	International Collaboration	n: 602 IG: 301 CG: 301	IG: 61.5 ± 9.33 CG: 61 ± 8.92	Letrozole	Immediate zoledronate 4 mg iv every 6 months	Delayed zoledronate 4 mg iv every 6 months	- **LS BMD** - TH BMD - Bone turnover biomarkers - Vertebral and nonvertebral fractures - Disease recurrence	36 months
Brufsky et al. (50) (Z-FAST)	*Cancer*	2012	International Collaboration	n: 602 IG: 301 CG: 301	IG: 61.5 ± 9.33 CG: 61 ± 8.92	Letrozole	Immediate zoledronate 4 mg iv every 6 months	Delayed zoledronate 4 mg iv every 6 months	- **LS BMD** - TH BMD - Bone turnover biomarkers - Vertebral and nonvertebral fractures - Disease recurrence	60 months
Bundred et al. (24) (ZO-FAST)	*Cancer*	2008	International Collaboration	n: 1065 IG: 532 CG: 533	IG: 57 (36-87) CG: 58 (37-81)	Letrozole	Immediate zoledronate 4 mg iv every 6 months	Delayed zoledronate 4 mg iv every 6 months	**- LS BMD** - TH BMD - Bone turnover biomarkers - Safety	12 months
Eidtmann et al. (47) (ZO-FAST)	*Ann Oncol.*	2010	International Collaboration	n: 1065 IG: 532 CG: 533	IG: 57 (36-87) CG: 58 (37-81)	Letrozole	Immediate zoledronate 4 mg iv every 6 months	Delayed zoledronate 4 mg iv every 6 months	**- LS BMD** - TH BMD - Vertebral and nonvertebral fractures - Disease recurrence - Overall survival - Safety	36 months
Coleman et al. (39) (ZO-FAST)	*Ann Oncol.*	2013	International Collaboration	n: 1065 IG: 532 CG: 533	IG: 57 (36-87) CG: 58 (37-81)	Letrozole	Immediate zoledronate 4 mg ev every 6 months	Delayed zoledronate 4 mg ev every 6 months	**- LS BMD** - TH BMD - Vertebral and nonvertebral fractures - disease recurrence - overall survival - safety	60 months
Llombart et al. (44) (E-ZO-FAST)	*Clinical Breast Cancer*	2012	International Collaboration	n: 522 IG: 252 CG: 270	IG: 58 (40-81) CG: 58 (44-78)	Letrozole	Immediate zoledronate 4 mg iv every 6 months	Delayed zoledronate 4 mg iv every 6 months	**- LS BMD** - TH BMD - Vertebral and nonvertebral fractures - disease recurrence - safety	12 months
Safra et al. (51) (NCT00376740)	*Oncology*	2011	Israel	n: 86 IG: 47 CG: 39	IG: 59.08 ± 8.5 CG: 61.18 ± 9.2	Letrozole following Tamoxifen	Immediate zoledronate 4 mg iv every 6 months	No treatment	**- LS BMD** - TH BMD - Vertebral and nonvertebral fractures - Disease recurrence - Overall survival	48 months
Takahashi et al. (43)	*Breast Cancer Research and Treatment*	2012	Japan	n: 194 IG: 97 CG: 97	IG: 61.47 ± 6.80 CG: 60.45 ± 6.56	Letrozole	Immediate zoledronate 4 mg iv every 6 months	Delayed zoledronate 4 mg iv every 6 months	**- LS BMD** - TH BMD - Bone turnover biomarkers - Vertebral and nonvertebral fractures	12 months
Hines et al. (36) N03CC (Alliance) trial)	*Breast Cancer Res Treat.*	2009	USA	n: 551 IG: 274 CG: 277	IG: 59.2 ± 11.20 CG: 59.6 ± 10.25	Letrozole	Upfront zoledronate 4 mg iv every 6 months	Delayed zoledronate 4 mg iv every 6 months	**- LS BMD** - TH BMD - FN BMD - Vertebral and nonvertebral fractures - toxicity	24 months
Wagner-Johnston et al. (37) (N03CC (Alliance) trial)	*Cancer*	2015	USA	n: 551 IG: 274 CG: 277	IG: 59.2 ± 11.20 CG: 59.6 ± 10.25	Letrozole	Upfront zoledronate 4 mg iv every 6 months	Delayed zoledronate 4 mg iv every 6 months	**- LS BMD** - TH BMD - FN BMD - Vertebral and nonvertebral fractures - toxicity	60 months

BMD, bone mineral density; CG, control group; FN, femoral neck; IG, intervention group; iv, intravenous; FN, femoral neck; LS, lumbar spine; sc, subcutaneous; TB, total body; TH, total hip; UK, United Kingdom; USA, United States of America. Primary outcomes of the study included were marked in bold.

Number of patients included ranged from 50 (41) to 3420 (34, 35) subjects. Seven RCTs (24, 36, 37, 39, 43, 44, 47, 48, 50–52) assessed participants who were treated with letrozole, 3 RCTs (41, 42, 45, 46) enrolled patients receiving anastrozole, one RCT (40) included patients treated with anastrozole or letrozole, and in 4 RCTs (29, 34, 35, 38, 49) patients were treated with anastrozole, letrozole, or exemestane.

BC patients received denosumab in 2 studies (26, 34, 35), zoledronic acid in 7 studies (24, 36, 37, 39, 43, 44, 47, 48, 50–52), risedronate in 3 studies (38, 45, 46), ibandronate in 2 studies (29, 41, 42), and alendronate in only one study (40). The comparator arm consisted in no treatment in two studies (46, 52), delayed treatment in 6 studies (24, 36, 37, 39–44, 47, 48, 50–52), and placebo in 7 studies (29, 34, 35, 38, 45, 49).

### Alendronate

From the studies included in this systematic review, only one assessed oral alendronate 5 mg in addition to calcitriol 0.5 µg daily in patients with EBC receiving adjuvant anastrozole or letrozole (40). The study showed significant differences between alendronate and placebo groups in terms of lumbar BMD (-0.5 ± 0.6% vs -3.5 ± 0.6%; p=0.05) at 24 weeks, whereas non-significant improvements were observed in hip BMD (-0.5 ± 0.4% vs -1.3 ± 0.5%; p>0.05). Diverse expression levels were only found in sCTx (72.4%; p<0.05), whereas osteocalcin (OCN) did not show significant differences between groups (29.0%; p>0.05) (as shown by **Table 3**).

**Table 3:** Main results of the articles included in the present systematic review.

Study	Fractures	LS BMD	TH BMD	FN BMD	Bone turnover biomarkers	Pain	Fatigue	Anxiety and Depression	Weakness	Lymphedema
***Alendronate***										
Rhee et al. (40)	NR	**24 weeks: -3.5 ± 0.6% vs -0.5 ± 0.6%; p=0.05**	**24 weeks: -1.3 ± 0.5% vs -0.5 ± 0.4%; p=NS**	NR	sCTx 24 weeks: 72.4%; p<0.05 OCN 24 weeks: 29.0%; p=NS	NR	NR	NR	NR	NR
***Denosumab***										
Ellis et al. (49)	4% vs 2% p=NR	**12 months: 5.5%; p<0.0001** 24 months: 7.6%; p<0.0001	12 months: p<0.0001 24 months: 4.7%; p<0.0001	12 months: p<0.0001 24 month: 3.6%; p<0.0001	1 month: sCTx 1 month: -9% vs -91%; p<0.0001 1 month: P1NP 1 month: -2% vs -29%; p<0.0001	Articular pain: 25% vs 24%; p=NR Back pain: 12.5% vs 14%; p=NR	14.2% vs 13.2%; p=NR	NR	NR	NR
Gnant et al. (34)	Incidence: 9.6% vs 5%; p=NR **Time to first fracture:** **HR 0.5 [95% CI** **0.39–0.65], p<0·0001**	12 months: -1.81% vs +3.94%; p<0.0001 24 months: -2.44% vs +5.85%; p<0.0001 36 months: -2.75% vs +7.27%; p<0.0001	12 months: -1.20% vs +2.67%; p<0.0001 24 months: -2.5% vs +3.70%; p<0.0001 36 months: -3.32% vs +4.60%; p<0.0001	12 months: -1.08% vs +2.22%; p<0.0001 24 months: -2.33% vs +2.86%; p<0.0001 36 months: -3.10% vs +3.41%; p<0.0001	NR	Articular pain: 26% vs 26% p=NS Back pain: 9% vs 9% p=NS Bone pain: 7% vs 8% p=NS	6% vs 6%; p=NS	NR	NR	NR
Gnant et al. (35)	Incidence: 9.6% vs 5%; p=NR **Time to first fracture:** **HR 0.5 [95% CI** **0.39–0.65], p<0·0001**	12 months: -1.81% vs +3.94%; p<0.0001 24 months: -2.44% vs +5.85%; p<0.0001 36 months: -2.75% vs +7.27%; p<0.0001	12 months: -1.20% vs +2.67%; p<0.0001 24 months: -2.5% vs +3.70%; p<0.0001 36 months: -3.32% vs +4.60%; p<0.0001	12 months: -1.08% vs +2.22%; p<0.0001 24 months: -2.33% vs +2.86%; p<0.0001 36 months: -3.10% vs +3.41%; p<0.0001	NR	Articular pain: 26% vs 26% p=NS Back pain: 9% vs 9% p=NS Bone pain: 7% vs 8% p=NS	6% vs 6%; p=NS	NR	NR	NR
***Ibandronate***										
Lester et al. (41)	No fractures	12 months: -3.19% vs +1.49%; p=0.012 24 months: -3.22% vs +2.98%; p=0.002	12 months: -2.27 vs +0.98; p=0.001 24 months: -3.90% vs +0.60%; p=0.003	NR	NTX 12 months: +39.5% vs -30.9%; p<0.001 sCTx 12 months: +34.9% vs -26.3%; p<0.001 bALP 12 months: +37.0% vs -22.8%; p<0.001	NR	NR	NR	NR	NR
Lester et al. (42)	3 vs 4; p=NR	60 months -2.88 vs 0.29%; p=NR	60 months 1.18% vs -3.71%; p=NR	NR	NR	NR	NR	NR	NR	NR
Livi et al. (29)	NR	12 months: -2.29% vs +2.96%; p=0.021 24 months: -4.22% vs +6.09%; p<0.0001	12 months: -2.35% vs +3.11%; p<0.001 24 months: -1.51% vs +4.64%; p=0.09	NR	NR	NR	NR	NR	NR	NR
***Risedronate***										
Greenspan et al. (38)	NR	12 months: -1.2% vs +2%; p<0.0001 **24 months: -1.7% vs +2.3%; p<0.0001**	12 months: -1.6% vs +0.5%; p<0.0001 **24 months: -2.7% vs +0.6%; p<0.0001**	24 months: 2.6 ± 0.8%; p=0.0009	sCTx 12 months: p<0.01 sCTx 24 months: p<0.01 P1NP 12 months: p<0.0001 P1NP 24 months: p<0.0001	NR	NR	NR	NR	NR
Markopoulos et al. (46)	No fractures	**12 months: 0% vs -0.4%; p=NS** 24 months: -1.5% vs +5.7%; p=0.006	**12 months: -1.3% vs 0%; p=NS** 24 months: -3.9% vs +1.6%; p=0.037	NR	NR	NR	NR	NR	NR	NR
Van Poznak et al. (45)	5 (2.1%)	**12 months: -1.2% vs +1.2%; p<0.0001** 24 months: -1.8% vs +2.2%; p<0.0001	12 months: -0.4% vs +0.9%; p=0.0023 24 months: -1.1% vs +1.8%; p<0.0001	NR	sCTx 6 months: +8.2% vs -44.0%; p<0.0001 sCTx 12 months: +6.1% vs -43.8%; p<0.0001 P1NP 6 months: -1.5% vs -41.8%; p<0.0001 P1NP 12 months: -2.4% vs -44.3%; p<0.0001 bALP 6 months: +1.6% vs -21.6%; p<0.0001 bALP 6 months: +3.9% vs -22.7%; p<0.0001	Articular pain: 7.8% vs 5.2%; p=NR Bone pain: 1.3% vs 1.3%; p=NR	NR	NR	No weakness	NR
***Zoledronate***										
Brufsky et al. (52)	2.1% vs 2.2%; p=NR	12 months: 5.2%; p<0.0001	12 months: 3.5%; p<0.0001	NR	NTX: 33.3%– 49%; p<0.0001 BSAP 30.3%– 48.9%; p<0.0001	Articular pain: 28.5% vs 31.7%; p=NR Back pain: 6.2% vs 5.6%; p=NR Bone pain: 5.9% vs 12.2%; p=NR	NR	Depression: 6.7% vs 3.9%; p=NR	NR	NR
Brufsky et al. (48)	6.3% vs 5.7% p=NS	**12 months: 4.3% p<0.0001** 24 months: 6% p<0.0001 36 months: 6.7% p<0.0001	12 months: 3.2% p<0.0001 24 months: 4.6% p<0.0001 36 months: 5.3% p<0.0001	NR	NTX: p=NS BSAP: p=0.0045	Articular pain: 37% vs 36.3%; p=NS Back pain: 10.7% vs 9.3%; p=NS Bone pain: 6.7% vs 13%; p=0.01	22.3% vs 26%; p=NS	Anxiety: 6% vs 4.7%; p=NS Depression: 11.7% vs 8.7%; p=NS	NR	5.7% vs 5.3%; p=NS
Brufsky et al. (50)	9.3% vs 11%; p=0.3803	48 months: p<0.0001 61 months: 8.9% p<0.0001	48 months: p<0.0001 61 months: 6.7% p<0.0001	NR	NTX: p=NS BSAP: p=0.0002	Articular pain: 47.0% vs 45.3%; p=NR; Back pain: 14.7% vs 17.3%; p=NR Bone pain: 16.0% vs 8.0%; p=NR Myalgia: 20.3% vs 15.7%; p=NR	33.7% vs 29.3; p=NR	Depression: 11.7% vs 14.0%; p=NR	NR	12.0% vs 10.0%; p=NR
Bundred et al. (24)	1.7% vs 1.5%; p=NR	**12 months: 5.7%; p<0.0001**	3.6%; p<0.0001	NR	BSAP 12 months: 45.6%; p<0.0001 NTX: 33%; p<0.0001	Articular pain: 29% vs 32.6%; p=NR Back pain: 6.5% vs 5.7%; p=NR Bone pain: 6.9% vs 12.3%; p=NR	11.4% vs 11.2%; p=NR	Depression: 5.3% vs 2.8%; p=NR	NR	NR
Eidtmann et al. (47)	6% vs 5% p=NS	**12 months: 5.27% p<0.0001** 24 months: 7.94% p<0.0001 36 months: 9.29% p<0.0001	NR p<0.0001	NR	NR	Articular pain: 40.7% vs 42.2%; p=NR Back pain: 11.4% vs 10.5%; p=NR Bone pain: 10.1% vs 15.3%; p=NR	15.1% vs 16%; p=NR	Depression: 6.5% vs 4.8%; p=NR	NR	5.4% vs 6.5%; p=NR
Coleman et al. (39)	NR	60 months: -5.4% vs +4.3%; p<0.0001	60 months: -4.2% vs +1.6%; p<0.0001	NR	NR	Articular pain: 46.9% vs 49% p=NR Back pain: 15.1% vs 15% p=NR Bone pain: 12.1% vs 18.5% p=NR	17.8% vs 17.7%; p=NR	NR	NR	NR
Llombart et al. (44)	1.9% vs 0.8%; p=NR	**12 months: 5.43%; p<0.0001**	12 months: 3.31%; p<0.0001	NR	NR	Articular pain: 38.9% vs 37.7%; p=NS Back pain: 7% vs 5.2%; p=NS Bone pain: 4.1% vs 8.3%; p=NS p<0.05 Shoulder pain: 5.9% vs 4%; p=NS	18.5% vs 15.1%; p=NS	Anxiety: 5.2% vs 3.6%; p=NS Depression: 5.6% vs 2%; p=NS	7.8% vs 9.1%; p=NS	4.1% vs 5.6%; p=NS
Safra et al. (51)	0 vs 0 p=NS	**24 months: 0.84; p<0.0001** **48 months: 0.76; p<0.0001**	24 months: 0.96; p=0.0041 48 months: 0.77; p=0.52	NR	NR	Articular pain: 26% vs 21%; p=NR	17% vs 8%; p=NR	4% vs 0%; p=NR	NR	NR
Takahashi et al. (43)	No fractures	**12 months: 4.9%; p<0.0001**	12 months: 4.4%; p<0.0001	NR	NTX 6 months: +21.8 vs -6.5%; p=NR NTX 12 months: +9.4% vs -23.6%; p=NR BSAP 6 months: +14.9% vs -33.6%; p=NR BSAP 12 months: +10.2% vs -39.4%; p=NR	Articular pain: 48.5% vs 51.6% p=NS	11,3% vs 9.6% p=NS	NR	NR	NR
Hines et al. (36)	NR	**12 months: 3.66%** **vs -1.66%; p<0.001** **24 months: 4.94% vs -2.28%; p<0.001**	12 months: 1.02% vs -1.41%; p<0.001 24 months: 1.22% vs -3.34%; p<0.001	12 months: 2.08% -0.09%; p<0.001 24 months: 3.36% vs 0.54%; p<0.001	NR	12 months: Back pain: 25% vs 23%; p=0.67 Myalgia: 7% vs 5%; p=0.53 Articular pain; 13% vs 11%; p=0.59	12 months: 5% vs 2%; p= 0.038	NR	NR	NR
Wagner-Johnston et al. (37)	25 vs 24; p= 0.84	**> 5% BMD differences:** **10.2% vs 41.2%; p<0.0001**	> 5% BMD differences in both TH and FN BMD: 7.6% vs 45.8%; p<0.0001		NR	NR	NR	NR	NR	NR

bALP, bone isoforms of alkaline phosphatase; BSAP, bone-specific alkaline phosphatase; CG, control group; C-telopeptide I (sCTx); FN, femoral neck; IG, intervention group; LS, lumbar spine; NR, not reported; NS, not significant; NTx, N-telopeptide; OCN, osteocalcin; P1NP, procollagen type I N-terminal peptide; TH, total hip. Primary outcomes of the study included were marked in bold.

### Denosumab

Three papers (2 studies) compared six-monthly denosumab 60 mg with placebo, reporting benefits in terms of fracture risk reduction or BMD improvement (34, 35, 49).

Gnant et al., in a collaborative study including 3420 patients, observed consistent differences in fracture incidence between patients treated with denosumab (5%) vs. untreated (9.6%) (34). Moreover, a significant difference in terms of time-to-first clinical fracture, the study primary endpoint, was observed between the two groups (HR 0.5, 95% CI 0.39–0.65, p<0.0001). Oppositely, the study by Ellis and colleagues (49) did not find major differences for fracture outcomes: no vertebral fractures were observed in both groups, the incidence of nonvertebral fractures was 6% in both arms, major nonvertebral fractures were observed in 3 women receiving denosumab (2%) and 5 women receiving placebo (4%).

Intriguingly, the two studies revealed significant differences between groups in terms of BMD. More in detail, Ellis et al. (49) reported significant differences between groups after 2 years of treatment (12 months: 5.5%; p<0.0001; 24 months: 7.6%; p<0.0001). On the other hand, hip BMD increased accordingly in both TH site (12 months: p<0.0001; 24 months: 4.7%; p<0.0001) and FN site (12 months: p<0.0001; 24 month: 3.6%; p<0.0001). Similarly, Gnant et al. (34, 35) underlined a significant difference between groups at 36 months (12 months: -1.81% vs +3.94%; p<0.0001; 24 months: -2.44% vs +5.85%; p<0.0001; 36 months: -2.75% vs +7.27%; p<0.0001). Hip BMD results were in line with the previous results with a significant increase in the denosumab group (12 months: -1.20% vs +2.67%; p<0.0001; 24 months: -2.5% vs +3.70%; p<0.0001; 36 months: -3.32% vs +4.60%; p<0.0001). Modifications in bone turnover were suggested by Ellis et al. (49), reporting significant differences between groups in C-telopeptide I (sCTx) and procollagen type I N-terminal peptide (P1NP), two markers of bone remodeling (1 month: CTX: -9% vs -91%; p<0.0001; P1NP: -2% vs -29%; p<0.0001). On the contrary, joint pain, back pain, bone pain and fatigue showed no differences when the two groups were compared. Outcomes are reported in detail in **Table 3**.

### Ibandronate

The effect of another anti-resorptive drug (i.e., ibandronate 150 mg every month) was assessed in BC survivors receiving anastrozole (41, 42) and anastrozole, letrozole, and exemestane (29). The study of Lester et al. in 2008 assessed the effects of Ibandronate (150 mg every month) for 24 months compared to placebo in osteopenic patients (41). On the other hand, patients with normal BMD did not receive any therapy while patients with osteoporosis received Ibandronate 150 mg every month. Interestingly, no fractures were recorded during the first 2 years (41). After 2 years, 3/20 patients continued to receive BPs over the next 3 years, while 8 patients received delayed ibandronate treatment. At 60 months, BMD changes were reported without reporting significant differences between groups (LS BMD: -2.88 vs 0.29%; p=NR; TH BMD: 1.18% vs -3.71%; p=NR). On the other hand, the study conducted by Lester et al. in 2012 recorded 4 fractures in the group that received ibandronate for 2 years, while the group treated with ibandronate after 2 years showed 3 fractures (42). In total, 10 fragility fractures were recorded: 4 fractures in the group treated with ibandronate for 2 years, 3 fractures in the placebo group treated with ibandronate after 2 years, and further 3 fractures in the osteoporotic group treated with ibandronate for 5 years.

Variations in lumbar and hip BMD were chosen as primary outcomes in both the ARIBON (41, 42) and BONADIUV trials (29). In both of them significant differences were found between ibandronate and placebo treated patients at both lumbar BMD and hip BMD after 12 and 24 months (29, 41). In particular, Lester et al. (41, 42) reported significant differences between groups in LS BMD (12 months: -3.19% vs +1.49%; p=0.012; 24 months: -3.22% vs +2.98%; p=0.002) and in TH BMD (12 months: -2.27 vs +0.98; p=0.001; 24 months: -3.90% vs +0.60%; p=0.003). Accordingly, Livi et al. (29) reported significant differences between groups (LS BMD 12 months: -2.29% vs +2.96%; p=0.021; 24 months: -4.22% vs +6.09%; p<0.0001; TH BMD: 12 months: -2.35% vs +3.11%; p<0.001; 24 months: -1.51% vs +4.64%; p=0.09).

Bone turnover biomarkers (sCTx, NTX, and bALP) were assessed instead only in the ARIBON study, with significant differences (NTX 12 months: +39.5% vs -30.9%; p<0.001; sCTx 12 months: +34.9% vs -26.3%; p<0.001; bALP 12 months: +37.0% vs -22.8%; p<0.001) (41). **Table 3** reported further details.

### Risedronate

The effects of risedronate 35 mg weekly in BC patients treated with anastrozole or letrozole, or exemestane were assessed in three studies (38, 45, 46). No fragility fractures were reported by Markoupolos et al. (46). In the study by Von Poznak et al., four patients in the control arm had fractures versus none in the risedronate arm (45). Lumbar BMD, a primary outcome in all these studies, was significantly increased in all trials after 24 months of treatment with risedronate (38, 45, 46). Similarly, significant differences were reported in hip BMD (38, 45, 46).

When bone turnover biomarkers were evaluated, significant differences between the risedronate and placebo groups were seen in the expression of isoforms of alkaline phosphatase (bALP), sCTx, N-telopeptide (NTX), and P1NP (38, 45). Joint pain was reported only by Van Poznak et al. only (45), without significant differences between groups (see **Table 3** for further details).

### Zoledronate

Seven studies (24, 36, 37, 39, 43, 44, 47, 48, 50–52) assessed the effects of endovenous administration of zoledronate 4 mg every 6 months in BC women treated with adjuvant letrozole. Of note, the study of Wagner-Johnston et al. evaluated EBC patients starting letrozole after completing tamoxifen treatment (37). Six studies (24, 36, 37, 39, 43, 44, 47, 48, 50, 51) compared the bone protection effect of immediate-start to delayed-start of zoledronic acid administration. On the other hand, Safra et al. (52) compared zoledronic acid administration with a control group not receiving any treatment.

In the delayed arm, zoledronic acid was initiated when BMD decreased to less than -2.0 or when a fragility fracture occurred. Although no differences were detected between the randomized groups regarding fracture incidence, significant effects in terms of both lumbar, the primary endpoint, and hip BMD increase were reported in the early administration group after 12, 24, 36, and 60 months (24, 36, 37, 39, 43, 44, 47, 48, 50, 51) (see **Table 3** for further details).

Bone turnover biomarkers were assessed in three studies, showing positive modifications in the early zoledronate group (24, 43, 48, 50, 51). Only one study did not record significant differences in sCTx concentrations after 36 months (48). Differences in terms of musculoskeletal pain, fatigue, anxiety, depression, weakness, and lymphedema were non-significant or not reported. **Table 3** summarizes the main results of these studies.

### Study Quality

Out of 21 studies included in this analysis, 20 of them (24, 29, 34–44, 47–52) were classified as high quality according to the Jadad scale (53). In particular, 6 papers (28.6%) (34, 35, 38, 40, 45, 49) obtained a score of 5, 1 paper (4.8%) (29) obtained score 4, 13 papers (61.9%) (24, 36, 37, 39, 41–44, 47–52) obtained a score of 3 and 1 paper (4.8%) (46) obtained a score of 1 (further details are depicted in **Table 4**).

**Table 4:** Quality assessment of the studies included in the present systematic review.

Articles	Domain					Score
	Random sequence generation	Appropriate randomization	Blinding of participants or personnel	Blinding of outcome assessors	Withdrawals and dropouts	
Brufsky et al. (52)	1	1	0	0	1	3
Brufsky et al. (48)	1	1	0	0	1	3
Brufsky et al. (50)	1	1	0	0	1	3
Bundred et al. (24)	1	1	0	0	1	3
Eidtmann et al. (47)	1	1	0	0	1	3
Coleman et al. (39)	1	1	0	0	1	3
Ellis et al. (49)	1	1	1	1	1	5
Gnant et al. (34)	1	1	1	1	1	5
Gnant et al. (35)	1	1	1	1	1	5
Greenspan et al. (38)	1	1	1	1	1	5
Lester et al. (41)	1	1	0	0	1	3
Lester et al. (42)	1	1	0	0	1	3
Livi et al. (29)	1	1	1	0	1	4
Llombart et al. (44)	1	1	0	0	1	3
Markopoulos et al. (46)	0	0	0	0	1	1
Rhee et al. (40)	1	1	1	1	1	5
Safra et al. (51)	1	1	0	0	1	3
Takahashi et al. (43)	1	1	0	0	1	3
Van Poznak et al. (45)	1	1	1	1	1	5
Hines et al. (36)	1	1	0	0	1	3
Wagner-Johnston et al. (37)	1	1	0	0	1	3

Points were awarded as follows: study described as randomized, 1 point; appropriate randomization, 1 point; subjects blinded to intervention, 1 point; evaluator blinded to intervention, 1 point; description of withdrawals and dropouts, 1 point.

## Discussion

AIs are considered the standard adjuvant therapy in postmenopausal women with early HR-positive BC (18, 19). However, the detrimental effect of AIs on bone health might significantly increase the risk of fractures, with negative consequences in terms of HRQoL and disability (54–56). Therefore, the implementation of tailored and effective interventions to reduce bone-related adverse events and preserve bone health is a crucial challenge in the complex management of patients with EBC receiving AIs. Thus, the present systematic review was aimed at summarizing the state of the art about bone-modifying agents to counteract Ais-induced bone loss, to provide data to guide the future research and clinical management of BC survivors.

Our findings pointed out the consistent improvement in BMD after 3 years of denosumab administration (34). Thus, denosumab could be considered among the most effective therapy to improve BMD and reduce fracture risk in EBC patients receiving AIs. Similarly, three RCTs provided long-term evidence (i.e., 5 years) about treatment with zoledronic acid, showing significant results in terms of lumbar and hip BMD improvement (37, 39, 51). Oral BPs also proved to be effective in enhancing BMD, even if the evidence supporting these drugs is weaker, given the smaller cohorts of patients, shorter treatment periods and less consistent results compared to those testing denosumab or zoledronic acid (29, 38, 40–42, 45, 46). Only the recent study from Livi et al. revealed a higher percentage of lumbar BMD improvement in BC survivors that were concomitantly treated with AIs and oral ibandronate compared with placebo (29). Yet, consistent data on the effectiveness of oral BPs on bone health in this setting are still lacking.

Interventions with anti-resorptive agents have also been found to have a positive impact on DFS. In particular, conflicting results were reported in the current literature with the ABCSG-18 trial (35) that underlined promising benefits of denosumab in DFS of post-menopausal early BC women receiving adjuvant aromatase inhibitor therapy. On the other hand, the D-CARE trial, which assessed the effects of denosumab in high-stage BC patients, did not report improvements in bone metastasis-free survival (57).

Similarly, controversial results were reported for BFs. In particular, the GAIN study showed no DFS benefits for both pre-menopausal and peri-menopausal BC patients who received oral ibandronate in the adjuvant treatment (58).

In accordance, large prospective studies assessing BPs failed to underline consistent effects on DFS endpoint in BC survivors (39, 51, 59) while positive data were provided by the EBCTCG meta-analysis reporting positive effects (RR for recurrence 0.86, 95% CI 0.78–0.94, p=0.002 in zoledronic acid arm) but restricted to postmenopausal women only (60). Therefore, to date, there is no consensus in terms of BPs prescription with the aim to improve DFS considering the large heterogeneous and discordant data.

On the other hand, a joint position statement of interdisciplinary cancer and bone societies suggested that adjuvant BPs should be considered in all postmenopausal women at risk for BC recurrence (61). Similarly, the Cancer Care Ontario and the American Society of Clinical Oncology (ASCO) guidelines recommended to consider BP prescription for all patients who are deemed at high enough risk of relapse (62). However, the authors underline that the lack of evidence did not allow a precise subgroups stratification for patients that might have major benefits from BP prescription (62).

Besides the role of BPs in overall and disease-free survival is still controversial, the cost-effectiveness of their routine use in clinical practice is far from being understood (63).

Taken together, these results suggest that the mechanisms underpinning the adjuvant effects of anti-resorptive drugs in patients with BC need to be further investigated.

Moreover, long-term effects of antiresorptive drugs also deserve to be considered. Although comprehensive management of AIs bone loss has been proposed to optimize bone health, to date, few evidence about the long-term effects of anti-osteoporotic treatments is available. International guidelines recommend the administration of anti-resorptive drugs for the whole duration of AIs therapy, but the optimal duration of these therapies is questionable (14, 64, 65). Moreover, it should be noted that AIs might be administered from 5 to more than 10 years (66), while studies assessing the long-term effects of denosumab or BPs in BC patients lasted 5-8 years (35, 39). Therefore, data supporting the long-term effects of anti-resorptive drugs on bone health in EBC patients receiving AIs are warranted.

This paper has some limitations which need to be taken into consideration. Firstly, only RCTs were included, thus excluding evidence provided by observational studies. Furthermore, because of statistical and methodologic heterogeneity among studies included, we did not carry out a pairwise or network meta-analysis.

In conclusion, bone health management is a cornerstone in the comprehensive management of patients with EBC receiving adjuvant AIs. Despite the remarkable advancements in understanding the mechanisms underpinning AI-induced bone loss, the optimal therapeutic framework for these patients remains a challenge for physicians.

This systematic review showed that denosumab and zoledronic acid might be considered the most effective anti-resorptive treatment options to improve BMD in patients with EBC on adjuvant AIs. However, robust data concerning the long-term effects of these drugs and their impact on the HRQoL are lacking. Thus, further studies addressing the long-term impact of these drugs are warranted. This could provide insightful evidence to guide clinicians in using tailored and effective treatments for BC survivors, to finally reduce their fracture risk and improve both HRQoL and long-term outcomes.

## Data Availability Statement

The raw data supporting the conclusions of this article will be made available by the authors, without undue reservation.

## Author Contributions

Study design and conceptualization: AS and MI. Databases searching: AS. Data screening: AS, LL, and MI. Data extraction: AS, LL, and MI. Data synthesis and interpretation: AS, LL, and MI. Manuscript drafting: AS and LL. Critical revision: KV, SM, NF, and MI. Visualization: ES, CCu, AA, and CCr. Study supervision: AS and MI. Study submission: AS. All authors read and approved the final version of the manuscript.

## Conflict of Interest

The authors declare that the research was conducted in the absence of any commercial or financial relationships that could be construed as a potential conflict of interest.

## Publisher’s Note

All claims expressed in this article are solely those of the authors and do not necessarily represent those of their affiliated organizations, or those of the publisher, the editors and the reviewers. Any product that may be evaluated in this article, or claim that may be made by its manufacturer, is not guaranteed or endorsed by the publisher.

## Supplementary Material

The Supplementary Material for this article can be found onlineat: https://www.frontiersin.org/articles/10.3389/fonc.2021.829875/full#supplementary-material
